# Oxytocin and serotonin in the modulation of neural function: Neurobiological underpinnings of autism-related behavior

**DOI:** 10.3389/fnins.2022.919890

**Published:** 2022-07-22

**Authors:** Feng Zhao, Hao Zhang, Peng Wang, Wenjie Cui, Kaiyong Xu, Dan Chen, Minghui Hu, Zifa Li, Xiwen Geng, Sheng Wei

**Affiliations:** ^1^Experimental Center, Shandong University of Traditional Chinese Medicine, Jinan, China; ^2^Key Laboratory of Traditional Chinese Medicine Classical Theory, Ministry of Education, Shandong University of Traditional Chinese Medicine, Jinan, China; ^3^TAIYUE Postdoctoral Innovation and Practice Base, Jinan, China; ^4^Chinese Medicine and Brain Science Core Facility, Shandong University of Traditional Chinese Medicine, Jinan, China; ^5^School of Environmental Science and Engineering, Shanghai Jiao Tong University, Shanghai, China; ^6^Department of Biology, Southern University of Science and Technology, Shenzhen, China

**Keywords:** autism, neural mechanisms, neural circuitry, oxytocin, serotonin

## Abstract

Autism spectrum disorders (ASD) is a group of generalized neurodevelopmental disorders. Its main clinical features are social communication disorder and repetitive stereotyped behavioral interest. The abnormal structure and function of brain network is the basis of social dysfunction and stereotyped performance in patients with autism spectrum disorder. The number of patients diagnosed with ASD has increased year by year, but there is a lack of effective intervention and treatment. Oxytocin has been revealed to effectively improve social cognitive function and significantly improve the social information processing ability, empathy ability and social communication ability of ASD patients. The change of serotonin level also been reported affecting the development of brain and causes ASD-like behavioral abnormalities, such as anxiety, depression like behavior, stereotyped behavior. Present review will focus on the research progress of serotonin and oxytocin in the pathogenesis, brain circuit changes and treatment of autism. Revealing the regulatory effect and neural mechanism of serotonin and oxytocin on patients with ASD is not only conducive to a deeper comprehension of the pathogenesis of ASD, but also has vital clinical significance.

## Introduction

Autism spectrum disorder (ASD) is a neurodevelopmental disease, which is generally diagnosed in the early growth and developmental stage of children. Its pathogenesis is complex and changeable. Genetic and/or environmental factors may both lead to the occurrence of autism, in which genetic factors play a leading role. At present, more than 100 gene mutations have been found to be closely related to the pathogenesis of ASD (Varghese et al., 2017). Autism patients are often accompanied by complex and diverse clinical symptoms. The main core symptoms are: impaired social communication, cognitive abnormalities, repetitive stereotyped behavior and limited interests (Geschwind, 2009). In addition to the above symptoms, ASD is diverse and heterogeneous. ASD Patients may be accompanied by epilepsy, sleep disorders, depression, anxiety and gastrointestinal dysfunction (Gorrindo et al., 2012; Maenner et al., 2012; Peters et al., 2014; Fulceri et al., 2016). According to the core typical symptoms, ASD is widely classified into several subtypes, including idiosyncratic autism, Rett syndrome (RTT), Asperger syndrome (AS) and fragile X chromosome syndrome (FXS; Benvenuto et al., 2009). In recent years, the incidence of ASD has increased gradually, which has attracted extensive attention from all walks of life. However, still now there are no unequivocal research results on the pathogenesis of the disease.

**FIGURE 1:**
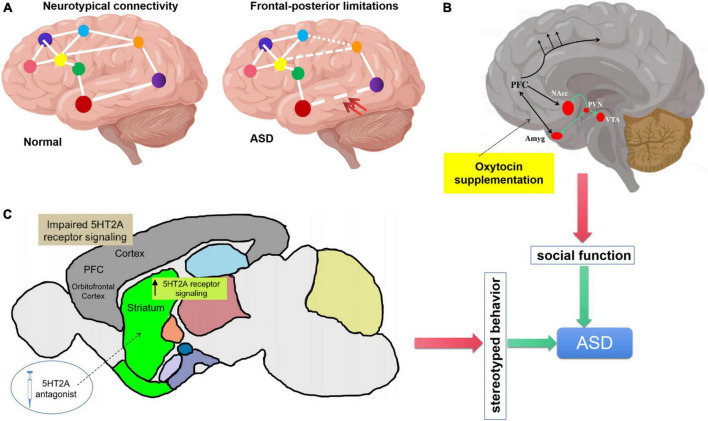
Regulation of oxytocin and serotonin on autism spectrum disorder (ASD) core symptoms. **(A)** Underconnecticity hypothesis, frontal-posterior underconnectivity in ASD (the double red arrow indicates) could explain the social cognitive impairment. **(B)** Schematic representation of oxytocinergic (green lines). OXT is synthesized in paraventricular nucleus (PVN), projects to key limbic sites (VTA, NAcc, and Amyg). **(C)** Cortico-striatal projections induce repetitive behavior. Elevation of serotonin 5HT_2A_ receptor signaling in the dorsomedial striatum gives rise to stereotypic behaviors. Application of serotonin 5HT_2A_ antagonist in the dorsomedial striatum also results in the rescue of repetitive behavior. 5HT_2A_, 5-hydroxy-tryptamine receptor 2A subtype.

Much of the earlier research on autism has paid close attention to behavioral changes, but judging ASD based solely on the criteria of presenting behavioral disorders often misses critical moments in brain development and loses the possibility of identifying the real cause. In recent years, the use of electroencephalography (EEG), functional magnetic resonance imaging (fMRI), diffusion tensor imaging (DTI), near-infrared optical imaging (fNIRS) and other scientific and technological technologies have provided substantial information for understanding the brain structure and functional changes of autistic patients (Vasa et al., 2016). Significant structural and functional changes have been found in brain regions such as the amygdala (Baron-Cohen et al., 2000; Phelps and LeDoux, 2005; Paul et al., 2010; Sato et al., 2017), medial prefrontal lobe (von dem Hagen et al., 2013; Martínez-Sanchis, 2014; Kim et al., 2016; Padmanabhan et al., 2017), basal ganglia, cerebellum, striatum, hippocampus, and hypothalamus (Carter et al., 2008; Ellegood et al., 2010, 2013, 2015; Peñagarikano et al., 2011; Portmann et al., 2014; Haberl et al., 2015; Wöhr et al., 2015; Allemang-Grand et al., 2017; Lauber et al., 2018) in studies of patients and animal models of ASD. Some hypotheses of neurodevelopmental abnormalities in the pathogenesis of ASD have also been formed, such as, abnormal synaptic development (Durand et al., 2007, 2012; van Spronsen and Hoogenraad, 2010; Clement et al., 2012), Neuronal excitability/inhibition disorder (Rubenstein and Merzenich, 2003; Graf et al., 2004; Peñagarikano et al., 2011; Sgadò et al., 2011; Chuang et al., 2014; Cochran et al., 2015; He et al., in press), dysfunctional connection of brain region (Figure 1A; Minshew and Keller, 2010; Sepulcre et al., 2010; Barttfeld et al., 2011; Just et al., 2012; Keown et al., 2013; Supekar et al., 2013), etc. In view of the non-univariate and complex causes, the genetic heterogeneity of ASD poses a significant obstacle to implement a unified therapeutic regime. Mechanism based treatments (Sahin and Sur, 2015) may have great potential, that is, to find a certain degree of commonness for the physiological changes that may be involved in the main symptoms of ASD, and then apply targeted intervention treatment. Considerable number of studies have demonstrated that oxytocin or serotonin treatment remedy cognitive and stereotyped behavior deficits in ASD patients or animal models.

In 1961, a study of 23 people with autism reported that six of them had abnormally high levels of serotonin in their blood (Cook and Leventhal, 1996). Since then, researchers have found that about a quarter of ASD patients have high levels of serotonin in their blood. Recent studies make it clear that anomalous serotonin levels are closely related to the occurrence of autism (McDougle et al., 1996; Chugani et al., 1999; Garbarino et al., 2019). Besides serotonin, oxytocin is also very important in the regulation of human central nervous system. As early as 1992, the studies of Modahl et al. (1998) found that the plasma oxytocin level in children with autism was lower than that in healthy children of the same age, and the increase of oxytocin level was not positively correlated with the increase of children’s age (Green et al., 2001). In recent years, the research on the etiology of autism at home and abroad is increasing. More and more studies show that the lack or underutilization of oxytocin level is related to its social disorder, and serotonin is related to stereotyped behavior. Therefore, oxytocin and serotonin can be used as a breakthrough to seek the treatment of autism and directly treat its core symptoms. In this review, we will discuss the possible neural mechanism and related brain regions of ASD, as well as the role of oxytocin and serotonin in the pathogenesis and treatment of ASD. The purpose is to summarize and elaborate the current research progress and correctly understand the application potential of oxytocin and serotonin in the treatment of ASD.

## Regulation of oxytocin on social function in autism spectrum disorder

The unknown etiology of autism leads to the difficulty of treatment. At present, the treatment is centered on the core symptom of social communication disorder, mainly through education and training, supplemented by drug treatment. Studies have shown that oxytocin can significantly improve social function. Oxytocin has been applied to the research field of autism as a possible treatment (Grace et al., 2018). Social communication is a complex process. The impairment of social communication in ASD patients is caused by social communication obstacles and language communication difficulties. Oxytocin can significantly improve the social communication ability of normal people. Oxytocin can strengthen the connection and trust between people and play the role of “social key” (Nave et al., 2015; Bernaerts et al., 2017). Oxytocin is a mammalian hormone, which can be secreted form neurons of hypothalamus, paraventricular nucleus and supraoptic nucleus, and is conducive to mammalian uterine contraction and lactation. Oxytocin is also very important in the regulation of human central nervous system. The plasma oxytocin level in children with autism was lower than that in healthy children of the same age, and the increase of oxytocin level was not positively correlated with the increase of children’s age (Modahl et al., 1998). Also in a meta-analysis shows that lower endogenous oxytocin level is excited in ASD children but not in adolescent or adult (Moerkerke et al., 2021). Anyway, oxytocin is generally considered to be closely related to ASD, especially ASD’s social defects (Cai et al., 2018; Zhou et al., 2021). In this regard, researchers suggest that the lack of oxytocin level may be one of the causes of social disorder in children with autism.

### Biological basis of oxytocin regulating social behavior in autism

Oxytocin receptors are distributed in brain regions involved in social behavior, including olfactory bulb, piriform cortex, amygdala and lateral septum (Figure 1B). Oxytocin plays an important role in the regulation of mammalian social behavior expression. Studies have shown that oxytocin receptor knockout mice show autism related behavior defects, while the supplement of exogenous oxytocin can improve this defect (Latt et al., 2018; Pati et al., 2020). Autism is associated with gene mutations such as Cntnap2 (the gene encoding contact associated protein like protein 2), intraperitoneal or intranasal injection of oxytocin can improve the social behavior defects of Cntnap2 gene ineffective mutant mice. The use of drugs to enhance the release of endogenous oxytocin can more effectively stimulate the oxytocin system and improve social abnormalities (Peñagarikano et al., 2015; Choe et al., 2022).

The human oxytocin receptor gene is located on chromosomes 3p25 and 3p26 (referring to the chromosome region), spanning 19 KB (KB represents 1000 bases), and contains 3 introns and 4 exons. Most alleles related to autism are located here. Some genome-wide linkage studies show that oxytocin receptor gene is a reasonable candidate gene for autism (Kelemenova et al., 2010; Lee et al., 2012). Referring to previous studies, oxytocin and its receptor gene can not only affect individual social behavior, but also have a close relationship with emotion and cognitive ability related to social behavior (Latt et al., 2018). In addition, studies have shown that abnormal oxytocin metabolism is significantly associated with social communication and communication disorders in individuals with autism. It is found that compared with the control group, the plasma oxytocin level of autistic children is lower, but its precursor level is higher, indicating that autistic children may be related to the difference of oxytocin processing in the brain, that is, there may be problems in the synthesis and processing of oxytocin in autistic children. It is precisely because of the abnormal synthesis and utilization of oxytocin that autistic children have social function defects (Zhang et al., 2012).

### Neural activity changes in autism spectrum disorder brain related with oxytocin

The role of amygdala in autism has been confirmed in many neuropathological and neuroimaging studies. Some studies suggest that there are inseparable relationship between anxiety and social disorder and amygdala dysfunction in patients with autism. The amygdala is located in the medial temporal lobe in front of the hippocampus, which is closely related to the social cognition and invasive behavior of autistic patients (Figure 1B). It plays a key role in emotional and social response (Satpute and Lieberman, 2006; Reetz and Dogan, 2020). The amygdala, as the main component of the cortical-striatum-thalamus-limbic cortical system and emotional circuit, participates in the process of stress emotional regulation and the formation of cognitive behavior. It has two specific functions, including eye gaze and facial processing (Britton et al., 2012). Amygdala lesions promote the formation of defense responses under stress stimulation (such as fear, aggression, emotional indifference and irritability in autistic children), and affect the memory regulation of emotional content and human eye gaze, thus affecting the social recognition and social communication ability of autistic patients. Animal studies have shown that oxytocin can reduce amygdala activity and reduce stress fear response. Studies on humans have shown that oxytocin can reduce the activation of amygdala and the functional connection with the upper part of brain stem (periaqueductal gray matter and its reticular structure), and reduce the individual’s experience of various negative emotions (Wójciak et al., 2012; Yoshida et al., 2014). Endogenous oxytocin can increase inhibitory neurotransmitters (γ- aminobutyric acid) release in central amygdala, decrease the activity of hypothalamic-pituitary-adrenal axis, in response to negative stress stimuli, thus improve the social anxiety of children with autism (Huber et al., 2005).

In addition to acting on the amygdala, oxytocin can also improve the corresponding sensory cortex in the brain of ASD patients to improve their perception of social information, and strengthen the functional connection between the cortex and the marginal reward system. As an important part of the default network, medial prefrontal lobe is not only considered as the core node of mental theory network, but also the neurophysiological basis of multi-channel perceptual integration (Martínez-Sanchis, 2014). Medial prefrontal lobe is widely used to explain the defects of autism spectrum disorders related to social ability, emotion, cognition and language. Within the default network, the resting functional connection between medial prefrontal lobe and precuneus lobe in autistic patients decreased (Padmanabhan et al., 2017). In the cognitive theory of mind task, autistic patients showed higher activation in areas such as medial prefrontal lobe and anterior cingulate gyrus (Kim et al., 2016). Using fMRI to observe the brain activity of ASD patients, it was found that after the intervention of nasal spray oxytocin, the activity of the right occipital gyrus, the left middle occipital gyrus and the left fusiform gyrus increased in ASD patients when viewing the social information of the human face. These brain regions were the early visual processing cortex in the brain. At the same time, it was also found that the cortical activity of the brain area around the right posterior superior temporal sulcus in ASD patients increased, and the functional connection between the prefrontal lobe (ventral prefrontal lobe and orbital frontal lobe) and the marginal reward system was strengthened (Figure 1B), which could improve the patients’ interest in social information perception and get happy rewards (Andari et al., 2016; Gordon et al., 2016).

**FIGURE 2:**
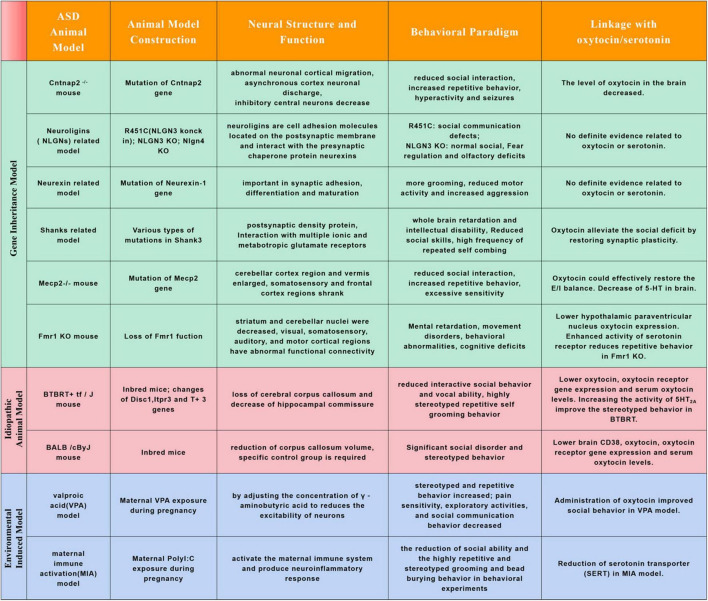
Commonly used animal models for autism spectrum disorders research and relations with oxytocin or serotonin. Autism spectrum disorder is a highly heterogeneous neurodevelopmental disorder. Its prevalence has been increasing in recent years, but the etiology and pathogenesis of the disease are not clear. It is an inevitable trend to explore its etiology and pathogenesis from animal models. The chart above summarizes the common ASD animal models and their characteristics, and linkage with oxytocin/serotonin.

Except in human, studies in ASD animal models also found neuroanatomical alterations in cortex (frontal, temporal cortical regions), basal ganglia, cerebellum, striatum, hippocampus and hypothalamus (Figure 2; Ellegood et al., 2010, 2013, 2015; Portmann et al., 2014; Haberl et al., 2015; Wöhr et al., 2015). In Mecp2 mutant mice, the cerebellar cortex region and vermis enlarged while the somatosensory and frontal cortex regions shrank, the change trends are similar to that of Rett syndrome patients (Carter et al., 2008; Ellegood et al., 2015; Allemang-Grand et al., 2017). In Mecp2 mutant, KCC_2_ deficiency may be one of the main causes of RTT syndrome, oxytocin could effectively restore the E/I balance in key brain regions under RTT pathophysiology by regulating the expression of KCC_2_ (Gigliucci et al., 2021). In Fmr1 KO mice, striatum and cerebellar nuclei were decreased, visual, somatosensory, auditory, and motor cortical regions also had abnormal functional connectivity (Ellegood et al., 2010; Haberl et al., 2015). In a recent study, Lewis et al. characterized ASD-related gene Fmr1 in parvocellular oxytocin neurons is essential parallel social information processing (Lewis et al., 2020). In addition, stimulation of protein kinase C epsilon could boost hypothalamic paraventricular nucleus oxytocin expression to normalize social and anxiety behavior in the FXS mice (Marsillo et al., 2021). In Cntnap2^–/–^ mouse model, there was a decrease in parvalbumin-positive interneurons in striatum resulting in altered activity of the cortico-striatal-thalamic circuit, and abnormal cortical migration during early developmental stage (Peñagarikano et al., 2011; Lauber et al., 2018). Cntnap2 was enriched in oxytocin neurons. The level of oxytocin in the brain of Cntnap2 knockout mice decreased, and the number of oxytocin neurons in the paraventricular nucleus (PVN) of the hypothalamus also decreased. The addition of exogenous oxytocin can greatly reduce the autistic social deficit of Cntnap2^–/–^ mice (Choe et al., 2022). In the Cntnap2^–/–^ mouse ASD model, it was found that the social behavior phenotype was mediated by the intestinal microbiota. *Lactobacillus reuteri* could reverse the number of oxytocin producing neurons, social defects and social interactions in VTA dopaminergic neurons (Buffington et al., 2021). In Shank3^–/–^ mouse model, oxytocin might alleviate the social deficit of ASD by promoting the expression of synaptic proteins and restoring synaptic plasticity (Harony-Nicolas et al., 2017; Reichova et al., 2020). In addition to these gene deficient ASD animal models, the indispensable role of oxytocin in regulating social behavior was also observed in spontaneous ASD animal models and environmental pollutant induced ASD models. Beta-carotene has been proved could rescue autistic-like social behavior in BALB/c and BTBR mice through enhancing brain oxytocin, oxytocin receptor gene expression and serum oxytocin levels (Bales et al., 2014; Avraham et al., 2019, 2021). Decreased oxytocin receptor (OXTR) and serum oxytocin levels were also reported in valproic acid (VPA) induced ASD models, and administration of oxytocin improved social behavior in these induced anminals (Hidema et al., 2020; Tsuji et al., 2021; Imam et al., 2022).

These studies on patients and animal models show that oxytocin is closely related to the exercise of social function. Most of the social defects of ASD are related to the decreased expression level of oxytocin or functional faultiness. Could additional oxytocin supplementation effectively treat ASD?

### Oxytocin supplementation can improve autism spectrum disorder symptoms

Autistic children lack the ability to understand emotions, thoughts and assumptions, and reduce prosocial behavior, resulting in various social and communication barriers, which become more obvious with age. Studies have shown that oxytocin users are more accurate in determining happiness expression and identifying all emotions faster and accurately than placebo users, suggesting that oxytocin has an impact on emotion regulation (Greene et al., 2018). A meta-analysis of the effect of intranasal oxytocin on emotion interpretation and expression showed that a single dose of intranasal oxytocin had no effect on the interpretation and expression of emotions in healthy people, but could improve their recognition of emotions, especially their sensitivity to fear (Nave et al., 2015; Bernaerts et al., 2017). The research on autistic patients also shows that the supplement of exogenous oxytocin can prolong the fixation time of patients to the eye area, increase eye contact and improve the emotional recognition ability of autistic patients to a certain extent (Watanabe et al., 2014). Previous animal experiments have shown that oxytocin plays a central role in animal social cognition. There is increasing evidence that oxytocin also takes a leading part in human social cognition, including reducing fear, increasing motivation and enhancing the significance of social information (Yatawara et al., 2016). The accuracy and response time of non-verbal information (i.e., information transmitted by expression) were calculated in 40 adult male ASD. After a single 24IU of nasal spray oxytocin treatment, ASD patients’ ability to understand non-verbal information increased. Oxytocin can enhance the cognitive ability of ASD patients to emotional rich contradictory information and strengthen the social communication between ASD patients and others. Oxytocin makes the communication of ASD patients more in line with the characteristics of normal people (Watanabe et al., 2014). Oxytocin induced neural signals are involved in the formation of social cognition in human ventromedial prefrontal cortex and regulate the activities in ventromedial prefrontal cortex to alleviate the social defects of autism (Guastella et al., 2015; Yatawara et al., 2016). Oxytocin regulates the processing of emotion related social information by increasing the eye gaze area, enhances the processing ability of facial stimulation, and infers the mental state of others from the eye gaze area, suggesting that oxytocin can improve the ability of autistic patients to deal with social cues (Quintana et al., 2017). For most species, oxytocin promotes social interaction and recognition of the same species, which may enhance social cognition by regulating the cortex that controls early olfactory processing (Zimmermann-Peruzatto et al., 2017; Liao et al., 2020). Early animal studies have shown that oxytocin can induce the prosocial behavior of kinship, the combination of maternal and offspring, and pairing. Oxytocin can also increase human prosocial behavior, such as trust behavior, cooperation behavior, intimacy behavior, mutual attraction and social adaptability, and emotional reactions in society, such as jealousy and disgust, motivation, communication and so on (Veening et al., 2015; Joushi et al., 2022). Oxytocin also plays the same role in autistic patients. For example, tests such as facial games and social interaction show that oxytocin can increase the trust and cooperation of autistic patients with peers (Auyeung et al., 2015; Owada et al., 2019).

In summary, oxytocin can significantly improve the social function of ASD patients, including the processing of social information, empathy and social communication ability. The brain regions of patients with ASD treated with oxytocin are mainly those closely related to these social functions, such as insula and amygdala related to empathy. The existing research results not only show the therapeutic effect of oxytocin, but also reveal the neural mechanism of brain abnormalities in patients with ASD, which makes people more deeply understand the disease of ASD.

## Abnormal serotonin is responsible for stereotypic behavior of autism

Serotonin (5-Hydroxytryptamine, 5-HT) is a bioactive amine substance existing in mammalian brain and surrounding tissues. It acts as a nutritional factor in the process of brain development. It is one of the earliest neurotransmitters in the central nervous system. Serotonin has many effects throughout the body, including mood, sleep, appetite and social interaction. In the gut, it stimulates the muscles involved in digestion. In the blood, it causes blood vessels to contract or dilate. In the brain, it transmits information between neurons. Its level in the brain is closely related to depression. Many antidepressants work by increasing serotonin levels at neuronal junctions (Kepser and Homberg, 2015). During early stage of brain development, 5-HT transporter (Serotonin transporter, SERT) dependent raphe nucleus-prefrontal cortex loop is associated with many neurodevelopmental disorders (Witteveen et al., 2013), including autism, depression, anxiety, obsessive-compulsive disorder and other neuropsychiatric diseases. In recent years, many studies have confirmed that the content of 5-HT in the serum of some patients with autism is high (Naffah-Mazzacoratti et al., 1993; Makkonen et al., 2011; Xiao et al., 2021). The change of 5-HT level affects the development of brain and causes behavioral abnormalities, such as anxiety, depression like behavior, reduced social ability and other symptoms. These symptoms are similar to the manifestations of autism.

### Abnormal 5-Hydroxytryptamine and the occurrence of stereotyped behavior

Prefrontal cortical, striatal and basal ganglia are tightly related with repetitive behaviors (Di Giovanni et al., 2006; Langen et al., 2012; Delmonte et al., 2013). Serotonergic 5HT_2A_ receptors mainly express in prefrontal cortical and striatal, the abnormal development of prefrontal lobe and striatum will produce repetitive stereotyped behavior. Increasing the activity of 5HT_2A_ in dorsomedial striatum can improve the stereotyped behavior of mouse model. The effect of 5HT_2A_ receptor antagonist on BTBR mice can improve their social behavior, cognitive ability, learning ability and repetitive modification behavior (Amodeo et al., 2016, 2017). 5HT_2A_ receptors are mainly located in glutamate neurons in frontal/parietal cortex and hippocampus, dopaminergic neurons in midbrain and endocrine cells in anterior pituitary. When activated, the 5HT_2A_ receptor either activates glutamatergic neurons in the frontal/parietal cortex, or inhibits the release of dopamine, or increases the release of neurohypophyseal peptide hormones such as growth hormone/luteinizing hormone/adrenocorticotropic hormone/prolactin. 5HT_2_ receptors expressed in different brain regions may play different functions. 5HT_2_ receptors expressed in different brain regions may play different regulatory functions. In the BTBR mouse model of autism, when 5HT_2A_ receptors antagonist (M100907) be added into the dorsomedial striatum reduces, such as grooming behavior and reversal learning deficits (Figure 1C). However, infusion of 5HT_2A_ receptor antagonist into the orbitofrontal cortex lead to excessive grooming behavior (Reiner and Anderson, 1990; Amodeo et al., 2017). In addition to 5HT_2_ receptors, there are 14 known subtypes of 5-HT receptors, and many receptors are related to the occurrence of autism. For instance, the hypomethylation of the promoter of human serotonin receptor 4 (HRT4) is an momentous marker for male ASD (Hu et al., 2020), serotonin receptor subtype 7 (5-HT_7_R) could be a therapeutic target for ASD (Lee et al., 2021), and serotonin 1A receptor expressed in the striatum also has been proved could regulate social behavior in ASD (Lefevre et al., 2020).

Serotonin levels in the blood are controlled in part by a protein called serotonin transporter. SERT is a transmembrane transporter with high affinity for 5-HT. It plays an important role in regulating the level of 5-HT (Rose’meyer, 2013). It can reuptake 5-HT in synaptic space. The level of 5-HT inside and outside cells depends on the level of SERT and the transcriptional activity of SERT (Gould et al., 2014). The level of SERT in the brain of patients with autism is abnormal. Nakamura et al. (2010) found that the SERT level in cingulate gyrus of adult patients with autism decreased through X-ray tomography. However, Oblak et al. showed that the decrease of SERT level in the brain of autistic patients was only reflected in the deep fusiform gyrus, while there was no significant change in the superficial or posterior cingulate gyrus (Oblak et al., 2013). SERT is encoded by the SERT gene (SLC6A4) located on chromosome 17q12. The variation of SLC6A4 gene is related to the formation of high 5-HT in the developmental stage of autism (Coutinho et al., 2004). A large amount of evidence suggests that SLC6A4 polymorphism regulates behavioral activity by maintaining central 5-HT levels (Jaiswal et al., 2015). In maternal immune activation (MIA) ASD animal model, reduction of histone deacetylase (HDAC) 1 and SERT were reported in hippocampal levels, which might induced the repetitive behaviors (Figure 2; Reisinger et al., 2016). Integrin-β3 gene can work together with SERT to regulate the level of 5-HT. Carter et al. showed that mice lacked Integrin-β3 can affect the level of 5-HT in brain and blood, which is manifested as social memory defect and repetitive stereotyped behavior (Carter et al., 2011). If SERT is absent or its activity is reduced, it will lead to the increase of extracellular 5-HT level and the decrease of intracellular 5-HT level, which will affect the normal function of 5-HT. Behavioral activity can be affected by changing the expression or activity of SERT (Carter et al., 2011).

### Abnormal serotonin level leads to other autism spectrum disorder syndrome

TRP, an essential amino acid for human, is a precursor of 5-HT. The decrease of TRP level in patients with autism causes the change of mitochondrial function, which affects synaptic plasticity, neuronal development and morphological development, and produces autistic behavior, such as mild depression, irritability and other symptoms (Rossignol and Frye, 2012). On the contrary, increasing TRP intake can reduce the symptoms of autistic patients. Acute TRP deficiency decreased the level of 5-HT in the brain of mice, accompanied by social behavior disorder, and the social behavior was improved after TRP supplementation (Daly et al., 2014). Abnormal TRP metabolism in patients with autism can affect metabolic pathways such as early brain development, mitochondrial balance and immune system activity. Then it leads to the abnormal development of neurons, especially in the frontotemporal lobe and limbic system, which may be the neuropathological factor of autism (Boccuto et al., 2013). TPH is the rate limiting enzyme in the synthesis of 5-HT, the expression of TPH2 gene decreased in the brain of patients with autism, and the defect of TPH2 gene will affect the shape of 5-HT neuronal circuit (Boccuto et al., 2013; Migliarini et al., 2013). A large number of animal experimental studies have found that the lack of TPH2 in the mouse brain may show social ability disorder, obsessive-compulsive behavior and cognitive impairment. The symptoms are similar to those of autism. The correlation between TPH2 and autism is also confirmed from the behavioral level (Schwartz, 2014).

ASD patients commonly have sensory dysfunction, which has been listed as one of the diagnostic criteria of autism (Marco et al., 2011). A large number of animal experiments have confirmed that 5-HT can affect the development of sensory function. Most of the studies are single sensory (visual, auditory and somatosensory), while there are few studies on multi sensory function. Gogolla et al. (2014) found that the multisensory integration ability in the insular cortex of BTRBT mice was impaired, and early administration of drugs could reverse this change. Multisensory function expands the integration of information through multiple sensory channels and can detect the connectivity of neurons. Using the Ala56 mouse model with SERT gene mutation, it is found that the disorder of 5-HT function in autism will affect the processing of multisensory and change the connectivity of neurons (Siemann et al., 2017). This experiment reveals the potential relationship between 5-HT, multisensory function and autism, and SERT take significant regulation on the development of sensory function, especially the somatosensory system. Sensory and multisensory networks are the basis for the formation of sensory perception and cognitive function, so sensory dysplasia will affect the formation of higher-level function. There are somatosensory abnormalities in patients with autism. In the developmental stage, improving the function of SERT can promote the development of sensory perception and prefrontal cortex. Therefore, it is feasible to improve the sensory function of patients with autism by changing the expression of SERT in presynaptic neurons or increasing SERT activity (Schauder et al., 2015).

## Limitations and future directions

### Oxytocin in the treatment of autism spectrum disorder

Oxytocin has been considered as a potential drug for the treatment of autism. Many studies have shown that animals lacking oxytocin receptors exhibit social dysfunction similar to autism. In many animal models of autism, exogenous administration of oxytocin can save the disorder of social function. To some extent, this also highlights the potential of oxytocin in the treatment of autism. At present, oxytocin as a drug is administered orally, intravenously and intranasally (Herscu et al., 2020). Oxytocin is degraded in the liver and gastrointestinal tract, so oral administration may not be very effective (King et al., 2009). Oxytocin is injected intravenously into the blood, and only a small part can enter the brain through the brain blood barrier, but this method is difficult to implement, which affects its wide use. One advantage of intranasal administration is that oxytocin can bypass the brain blood barrier and reach the cerebrospinal fluid within 30 min (Reddihough et al., 2019). This means that intranasal administration allows oxytocin to reach the brain directly without systemic side effects on other organs of the body.

There have been many clinical trials of oxytocin in the treatment of autism, but some research results show that oxytocin has no or almost no effect on patients. For example, in a study of 19 adult autistic patients, six weeks after intranasal administration, there was no improvement in patients’ severe repetitive behavior. In another trial of 38 adolescent boys, those who took oxytocin (intranasal administration) did not improve their emotional recognition and social skills compared with the control group. There are also many other experiments suggesting that oxytocin has no effect on social function in patients with ASD (Welch et al., 2014; Kablaoui et al., 2018). In a study of 355 children and adolescents with ASD who were given intranasal oxytocin continuously, there was no significant difference in cognitive behavior between the treatment group and the placebo group within 24 weeks (Geschwind, 2021; Sikich et al., 2021). This may be because oxytocin is also affected by other factors when interfering with ASD patients, and there is great heterogeneity among ASD patients. The intervention effect of oxytocin on ASD patients can not be generalized. For example, oxytocin can interact with dopamine, γ-aminobutyric acid, glutamate, norepinephrine and acetylcholine to promote the generation of human social learning model and improves the ability of attention, association, learning and memory (Lee et al., 2020). Oxytocin receptors also exist in the key hubs of the autonomic nervous system (brainstem solitary nucleus and hypothalamus), suggesting that they are related to the mediation of human emotion, social motivation, social learning and memory and sympathetic pathways (Dadds et al., 2014).

The research results of oxytocin in the field of ASD treatment are still controversial and need more basic and clinical data support. Fortunately, some heavy studies in recent years have shown the importance of oxytocin in regulating the social ability of autism. Fortunately, some heavy studies in recent years have shown the importance of oxytocin in regulating the social ability of autism. For example, Katrina et al. Used two complementary but independent whole brain imaging methods of mouse resting fMRI and c-Fos-iDISCO + imaging to construct the whole brain activity and connectivity map of Cntnap2 knockout (KO) mouse model, established the loop and system level mechanism of social defects in Cntnap2 KO mice, and revealed that the nucleus accumbens (NAC) is a region that can be regulated by oxytocin (Choe et al., 2022). Researchers from Peter scheiffele’s team at the University of Basel, Switzerland, recorded the activity of dopamine neurons in the ventral tegmental area brain slices of nlgn3 deficient mice and found that oxytocin injection into the slices increased the neuronal discharge of control mice, but did not increase the neuronal discharge of knockout mice. Finally, it was found that the loss of Nlgn3 affected the synthesis of proteins in these dopamine neurons, thus affecting the response of neurons to oxytocin (Hörnberg et al., 2020). According to these studies, more systematic research is needed on the mechanism of oxytocin in brain nerve development, circuit establishment, function exercise and ASD in the future, so as to give full play to its most potential role in the treatment of ASD.

### Challenges and potentiality of serotonin in the treatment of autism spectrum disorder

A series of drugs used to treat depression, anxiety and obsessive-compulsive disorder seems to alleviate the compulsive behavior in adults with ASD. These drugs, known as serotonin reuptake inhibitors (SRI or SSRI), regulate human physiology by increasing the level of the neurotransmitter serotonin in the brain, including fluoxetine (Prozac) and citalopram (Celexa). Preliminary evidence suggests that in adults with autism, the active ingredient of the drug “stimulant” can increase serotonin levels in the brain, which seems to alleviate social anxiety. In some animal experiments, it has also been shown that serotonin is closely related to the abnormal behavior of ASD. A significant decrease in brain of 5-HT level was also observed in Mecp2-/- mice and RTT patients. Some studies have shown that fluoxetine can improve brain 5-HT level, thus slowing down stereotyped behavior in ASD animals (Villani et al., 2020, 2021). FPT, a partial agonist of serotonin receptor, reduces repetitive behaviors in Fmr1 knockout mouse model od ASD (Armstrong et al., 2020). However, recently published studies show that these drugs do not seem to significantly reduce repetitive behavior in autistic children (Hua et al., 2018; Choe et al., 2022). Does these studies show that these drugs are useless in the treatment of autism? Or did they reveal problems with the way drugs are tested?

The role of SSRI is to block the serotonin transporter and prevent the reabsorption of serotonin into neurons, resulting in the increase of serotonin concentration in the synaptic cleft. So SSRI intervention to increase the serotonin in the synaptic cleft of autistic children should improve the stereotyped behavior and other ASD symptoms. Unfortunately, SSRI treatment has been reproted to be effective for adults, but it does not significantly improve the symptoms of autistic children and adolescents (Williams et al., 2013; Wichers et al., 2019). At present, it is not clear why this significant difference in therapeutic effect exists, and it may be related with the therapeutic dose of SSRI. For autistic children and adolescents, due to the lack of research evidence, we should give priority to behavioral therapy, and only consider issuing SSRI when they are clearly diagnosed with diseases that respond to SSRI, such as, depression and anxiety usually accompanied by autism. At the same time, the use of SSRI also requires caution, because the use of SSRI by pregnant mothers increases the risk of ASD in young children (Andalib et al., 2017), and the role of SSRI in improving adult ASD is controversial (Katrina et al., 2013; Leshem et al., 2021).

In conclusion, serotonin may be helpful in the treatment of repetitive behavior or social disorder in autism, but further research is needed. At present, some researchers are testing whether drugs that activate serotonin receptors will make autistic mouse models more social (Armstrong et al., 2020). Other researchers are studying strategies to inhibit serotonin transporter activity without completely blocking it (Robson et al., 2018). For example, treatment of p38αMAPK inhibitor could normalized hippocampal 5-HT clearance and ameliorate core and comorbid phenotypes present in ASD models (Robson et al., 2018). This is also one of the future directions of serotonin and autism research.

### Other perspectives in autism research

At present, the etiology of autism is not clear, both genetic factors and environmental factors. Some studies have shown that intestinal flora contributes to the pathogenesis of autism. Wang Juan group found that there were obvious defects in detoxification enzymes and pathways in children with autism. The impaired detoxification function of intestinal microorganisms led to toxin accumulation and mitochondrial dysfunction, which is the core component of the pathogenesis of autism. The research team collected fecal samples from 39 children diagnosed with autism and 40 children without the disease, and sequenced each fecal sample. It was found that the proportion of detoxifying enzymes in children with autism was different from that in children without autism. It is speculated that due to the impact of intestinal flora on intestinal detoxification process, Children may develop autism. In turn, this allows environmental toxins to enter the blood, damaging mitochondria in brain cells, leading to autism related symptoms (Zhang et al., 2020). At the same time, another studie had reported that *L. reuteri* treatment can selectively save the social defects in genetic, environmental and idiopathic ASD models. *L. reuteri* plays a role in a vagus nerve dependent manner and saves the social interaction induced synaptic plasticity in the ventral tegmental area of ASD mice (Sgritta et al., 2019). However, Yap et al. (2021) believe that there is no direct link between intestinal flora and autism. The difference of intestinal flora between autistic children and normal children is due to the decline of dietary diversity and narrow dietary types caused by autistic symptoms, which leads to the decrease of intestinal flora diversity, leading to constipation and gastrointestinal symptoms. At present, there is no definite conclusion in relevant research fields.

In addition to studying the pathogenesis of ASD with the help of traditional rodent models, the application of brain like organs may reveal the essence closer to human pathogenesis. Recently, Paola Arlotta team from Harvard University used the human cortical organoid model to identify that mutations in three ASD risk genes from different donors – Suv420h1, Arid1b and Chd8 can lead to two main cortical neuron lineages γ- GABAergic neurons and deep excitatory projection neurons develop abnormally, but the degree of expression is affected by the individual genomic environment. Calcium imaging in organoids shows abnormal neural circuit activity after early developmental changes (Paulsen et al., 2022). This study reveals cell type specific neurodevelopmental abnormalities shared among ASD risk genes and closely regulated by the human genome environment, and explains how different risk genes contribute to the phenotypic characteristics of ASD.

For now, the research on the pathogenesis and treatment of ASD is in full swing. It is believed that with the help of new technologies such as single-cell sequencing, high-precision imaging and brain like organs, the treatment methods of ASD in Colleges and universities can be found in the future, which will bring glad tidings to patients and society.

## Author contributions

FZ and SW wrote the review manuscript. All authors participate in the conception and design of this review and contributed to the article and approved the submitted version.

## Conflict of interest

The authors declare that the research was conducted in the absence of any commercial or financial relationships that could be construed as a potential conflict of interest.

## Publisher’s note

All claims expressed in this article are solely those of the authors and do not necessarily represent those of their affiliated organizations, or those of the publisher, the editors and the reviewers. Any product that may be evaluated in this article, or claim that may be made by its manufacturer, is not guaranteed or endorsed by the publisher.
